# Social Frailty and Social Isolation in the Context of Dementia: A Simultaneous Concept Analysis

**DOI:** 10.1002/gps.70074

**Published:** 2025-04-19

**Authors:** Ziyue Wang, Dympna Casey, Duygu Sezgin

**Affiliations:** ^1^ School of Nursing and Midwifery University of Galway Galway Ireland

**Keywords:** dementia, nursing, simultaneous concept analysis, social frailty, social health, social isolation

## Abstract

**Objectives:**

Early management of risk factors related to social health such as social frailty and social isolation could modify the progression of dementia and reduce its impact on people with dementia. However, due to overlapping definitions and interchangeable use of measurement tools, the precise distinction between these two concepts is unclear. This simultaneous concept analysis aimed to examine the definitions and interrelationships between the concepts of social frailty and social isolation within the context of dementia.

**Methods:**

The simultaneous concept analysis method developed by Haase et al. was employed. A literature search was conducted across six databases (Ovid Medline, CINAHL, PsycINFO, Scopus, Embase and Cochrane Library) to retrieve original research, review and theoretical papers, published in English. Data from the literature was synthesised and analysed thematically following Braun and Clarke's six steps.

**Results:**

The attributes of concepts of social frailty and social isolation overlap, including being alone and having limited or less social activities, engagements or interactions. The specific attributes of social frailty are financial difficulties and less talk while social isolation is distinctly marked by a lack of social networks or social relationships. Socio‐economic welfare is a specific antecedent of social frailty, while decreased access to social resources and lower social well‐being are distinctive consequences of social isolation. Cognitive decline and dementia are distinctive antecedents of social isolation; however, they also exist as shared consequences of both social frailty and social isolation.

**Conclusions:**

This simultaneous concept analysis clarified the similarities and differences between social frailty and social isolation in the context of dementia. A clearer understanding of the interrelationships between social frailty and social isolation and distinct and overlapping characteristics of them will support strategies to comprehensively address social health issues experienced by people with dementia.

**Summary**

- Although there are overlaps between the attributes of both concepts, social frailty is a less mature concept in the area of dementia than social isolation.
- Social frailty focuses on the quantity of engaging in social activities or interactions, whereas social isolation encompasses a broader range of measurements, focussing on both the quantity and quality of social connectedness, not only social interactions. Social frailty might indicate a one‐way reduction of activities such as ‘visiting friends less’ due to the reasons related to people with dementia. On the other hand, social isolation refers to a reduction or absence of two‐way activities highlighting a broader impact extending beyond socially isolated individuals' actions.
- Attributes of social frailty distinctly reflect an absence of social resources and financial difficulty, while decreased access to social resources and having lower social well‐being are distinctive consequences of social isolation. This suggests a relationship between the two concepts. Cognitive decline and dementia symptoms and diagnosis are antecedents for social isolation. On the other hand, cognitive decline and increased risk for dementia are recognised as consequences of both social frailty and social isolation.
- This concept analysis provides an improved understanding of the distinctions and overlaps between social frailty and social isolation, which can help development of targeted interventions to address specific social needs and improve social health outcomes in dementia population.

## 1 Introduction

The onset age of dementia is occurring significantly earlier [1, 2], and the prevalence of dementia is increasing [3]. The impact of dementia on individuals extends beyond cognitive decline, significantly affecting their social health [4]. The lack of dedicated social health services for people with dementia leaves inadequate support for managing the social aspect of their condition, leading to unmet needs related to their social health [5]. Taking cognisance of the unique social health issues and needs of people with dementia could enhance their overall care experiences and health outcomes [6].

Huber et al. [7] proposed a reconfigured definition of health, highlighting the social domain alongside the physical and mental domains. Social health, described as an umbrella concept [8], includes the capacity to fulfil potential and obligations, the ability to manage life with some degree of independence and participation in social activities [7]. Social frailty, usually portrayed as someone who lives alone, is unable to provide help to others, goes out less frequently, visits friends infrequently and does not talk to anyone on a daily basis [9], and social isolation serve as markers of social health [8]. Studies have shown that both social frailty and social isolation are linked to cognitive decline [10, 11, 12] and the onset of dementia [13, 14, 15]. Moreover, social frailty can increase the risk of adverse outcomes for individuals with dementia [16, 17]. Similarly, social isolation is highly associated with the severity of dementia [18] and increases physical and mental health risks for dementia [13]. The literature demonstrates that social frailty and social isolation are critical factors that could potentially increase the challenges faced by individuals with dementia. It is crucial therefore to investigate the factors contributing to social frailty and social isolation to devise preventive measures and interventions for individuals with dementia, to enhance their social health.

Notably, the terms social frailty and social isolation are sometimes used interchangeably [19, 20], which indicates an overlap in their meanings within the dementia context [21]. Social frailty is defined by Bunt et al. [22] as ‘*a continuum of being at risk of losing or having lost, resources that are important for fulfilling one or more basic social needs during the life span*’. According to Broese [23], social frailty encompasses insufficient participation or no participation in social networks and the perception of a lack of contacts and support. Conversely, social isolation is seen as a lack of social contacts and interactions with family members, friends or the community [24]. Despite shared elements, there is ambiguity regarding whether social frailty and social isolation bear identical meanings, as social isolation could be interpreted as a component of social frailty. Therefore a clear understanding of each concept and the exact interrelationship between them is warranted [25, 26].

Identification of social frailty and social isolation in people with dementia and in general population involves the use of measurement tools; however, the items listed in these tools often share overlapping criteria. Bessa et al. [27, 28] examined 27 tools that measure social frailty. They reported that most questions to assess social frailty refer to its social domains and include living alone, reduced social networks, lack of social support, loneliness and infrequent social activities [27, 28]. However, the item ‘loneliness’ is considered a subjective construct within the definition of social isolation [29]. Another example of this overlap is that several items in the Social Isolation Score [30] used to measure social isolation, such as the items ‘living alone’, ‘contacting friends or neighbours’ and ‘talking to friends or neighbours’, share elements with Makizako et al. [9]'s measurement tool of social frailty. In a study by Díaz Alonso et al. [31], conducted with a European sample, items reflecting social isolation [24] such as infrequent contact with family, friends and neighbours were used to measure social frailty. In other examples of studies conducted in Asian countries, the Lubben Social Network Scale‐6, which is widely used to assess social isolation [32], was used to measure social frailty [19, 20]. These examples demonstrate that the items used in the respective tools to measure social frailty and social isolation overlap and are used interchangeably. There is a need thus to examine each concept carefully and identify the similarities, differences and interrelationships between them. This will contribute to differentiating the meaning of these concepts as well as clarifying their respective measurement tools. The overlap in current measurement tools for social frailty and social isolation is noted, but in the context of dementia, there is no evidence of how these concepts are understood or what precise interrelationship exists.

Simultaneous concept analysis is a strategy used to explore two or more concepts to achieve a greater comprehension of each concept and their interrelationship [33]. Through a detailed analysis of the antecedents, attributes, and consequences of social frailty and social isolation, a better understanding of their definitions can be ensured. Having a clear understanding of these two concepts will help researchers and clinicians identify the socially frail and socially isolated individuals with dementia, to address their specific needs. Furthermore, this level of understanding will enhance the design of interventions and care practices, leading to improved healthcare outcomes and quality of life for both socially frail and socially isolated people with dementia.

## 2 Methods

### 2.1 Aim

This simultaneous concept analysis aimed to examine the concepts of social frailty and social isolation within the context of dementia. This analysis identified distinguishing, overlapping and similar attributes, antecedents and consequences for each concept and explored their interrelationships simultaneously.

### 2.2 Study Design

This study employed simultaneous concept analysis (SCA) using a framework developed by Haase et al. [33, 34], based on Rodgers’ evolutionary concept analysis methodology [35]. This simultaneous concept analysis was registered on the Open Science Framework [36]. The analysis involved nine steps [33], which are described in detail below:

#### 2.2.1 Step 1‐Development of the Consensus Group

The consensus group comprised three researchers (Z.W., D.C., D.S.), whose research focuses on dementia and social frailty. The core consensus group (Z.W., D.S.) met biweekly to discuss the concept analysis process including the development of the SCA strategy, refined definitions of each concept, identification of the specific elements of each concept, and their sequence of occurrence and clarification of interrelationships. The full consensus group met every 6 weeks to review the process over 7 months.

#### 2.2.2 Step 2‐Selection of Concepts to be Analysed

With the aim of this simultaneous concept analysis, two concepts, named social frailty and social isolation, were selected following comprehensive discussions between three researchers (Z.W., D.C., D.S.). The analyses were conducted for each concept simultaneously in the context of dementia.

#### 2.2.3 Step 3‐Refinement of the Concept Clarification Approach

A scoping review of the literature was performed. Specific elements of this approach are described below:

##### 2.2.3.1 Data Source

The literature searches were performed on six databases: Ovid Medline, CINAHL, PsycINFO, Scopus, Embase and Cochrane Library.

##### 2.2.3.2 Literature Search

The literature search followed the guidelines for scoping review [37]. A pilot search on PubMed was conducted to identify all terms related to the two concepts of social frailty and social isolation as well as dementia. The MeSH terms ‘Social Isolation’, ‘Dementia’ and ‘Alzheimer Disease’ were identified. However, there were no relevant MeSH terms for ‘social frailty’. The final literature search strategy was refined with the assistance of a librarian. The final search was conducted in December 2023. All searches were limited to English, without a time limit. Detailed search strategies for all databases and preliminary search results are presented in Supporting Information S1: Appendix 1.

##### 2.2.3.3 Eligibility Criteria

According to the PRISMA‐ScR [37] guidelines, the publications were screened using the following inclusion criteria (1) provide conceptual or operational definitions or descriptions of social frailty, or social isolation, (2) address the topic within the context of dementia, (3) reporting from the perspectives such as various contexts including different care settings (e.g., community, residential care or acute care), diverse populations, and professional background of authors, (4) published in peer‐reviewed journals following any study design including review articles, editorials, and commentaries and (5) published in English. Following a semantic approach, publications were included if they defined the terms social frailty or social isolation. Publications excluded were those that (1) include social frailty or social isolation but did not provide a definition or description, (2) were not in the context of dementia, (3) were not published in peer‐reviewed journals, (4) were only conference paper, (5) were not available in English, (6) focussed on social frailty or social isolation only in caregivers of people with dementia considering that this is not the scope of this SCA, (7) were protocol registrations on an online platform or (8) were not available in full‐text.

##### 2.2.3.4 Literature Selection

The search results were imported to Endnote, and afterwards to Rayyan for de‐duplication and systematic screening. A random selection of publications (10% of the search results) was piloted by three researchers for the screening of titles and abstracts (Z.W., D.S., H.Z.). The conflicts between the researchers were resolved by discussions and consensus. This ensured the clarification and refinement of the inclusion and exclusion criteria. After that, two researchers (Z.W. and H.Z.) completed the title and abstract screening for the remaining publications. For the full text screening, a random selection of 10% of the publications were screened by two researchers (Z.W., D.S.), and the remaining publications were screened by one researcher (Z.W.). In case of unclarity regarding inclusion of a full text publication, discussions were held between two researchers (Z.W., D.S.) and decisions were made by consensus.

##### 2.2.3.5 Data Extraction

Data extraction was carried out following the data extraction form developed by Z.W. and D.S., including (1) title, the year of publication, author (s)’ names and their disciplines; (2) design of the publication; (3) aims of the publication; (4) population/participant characteristics, setting, assessment tools (if any); (5) conceptual definitions, operational definitions and narrative descriptions by people with dementia as reported; (6) factors identified as antecedents of the concepts; (7) identified consequences of the concepts; (8) concepts identified by authors as relevant to the concepts of interest; (9) ‘alternative terms’ used by authors to refer to the concepts; (10)any examples (such as cases) of social frailty or social isolation provided by authors; (11) information reported as the primary or secondary source; (12) other notes. Data was extracted from the included publications by Z.W. and the data was reviewed by DS for accuracy.

#### 2.2.4 Step 4‐Clarification of Individual Concepts

According to Haase et al. [33], the purpose of data analysis is not to develop a new, complete or finished definition of concepts, but rather to provide clarification of each concept. A thematic analysis was performed as suggested by Haase et al. [33] to examine the concepts of social frailty and social isolation to strengthen the characterisation and clarification of each concept. Braun and Clarke's six steps [38, 39] were followed for the thematic analysis. The extracted data was categorised into the groups of conceptual definitions, operational definitions and narrative descriptions. The data was first read and coded by Z.W. [33, 40]. As part of this process, separate coding manuals were developed for attributes, antecedents and consequences. Another researcher (D.S.) then reviewed this coding and consensus was reached on the coding. The codes were collated and categorised into draft constructs for each concept by Z.W. and D.S. The constructs, which represent themes in the thematic analysis process, were then compared with each other to identify the common factors and distinctive attributes, antecedents, and consequences of both concepts. The term ‘distinctive’ was used to describe an attribute, antecedent or consequence that is present in only one of the two concepts but not both, and therefore played a crucial role in differentiating one concept from another.

#### 2.2.5 Step 5‐Development of Validity Matrices

Validity matrices were constructed for attributes, antecedents and consequences (Z.W.) through the identification of common factors and an examination of the connections between concepts to attain theoretical coherence. During this iterative process, Z.W. and D.S. interrogated each element, identified inconsistencies and gaps and made revisions as needed until all the elements were identified within the context of dementia.

#### 2.2.6 Steps 6 and 7‐Revision of Individual Concept Clarification and Re‐Examination of Validity Matrices

The consensus group convened to review the previous classifications of concepts and make any required revisions. Additionally, the group engaged in discussions, examinations and re‐evaluations of the concept analyses and validity matrices. Taking into account each element, considering others through constant comparisons to clarify the interrelationships and differences between the two concepts.

#### 2.2.7 Step 8‐Development of a Process Model

After a consensus on attributes, antecedents and consequences were more clearly delineated a process model was developed [33]. The model examined the similarities and differences across concepts, and the interrelationships across both concepts and processes throughout the model.

#### 2.2.8 Step 9‐Submission of the SCA Results to Peers for Critique

After exploring the concepts of social frailty and social isolation, the simultaneous concept analysis results were shared with peers, such as dementia researchers (*n* = 14), clinical practitioners in dementia care (*n* = 14) for example, staff at memory clinics and memory assessment support services and a Patient and Public Involvement (PPI) contributor (a family caregiver of a person with dementia), by presenting the protocol and the preliminary results in meetings, seminars, and conferences to obtain their input, feedback and an objective evaluation of the findings. They were contacted via the TeamUp for Dementia Research (TUDR) of the Alzheimer Society of Ireland (TUDR is a service where people living with dementia and their families can register their interest in involving in dementia research). The researchers working in dementia and clinical practitioners were identified from a local hospital and community care services in Ireland, Psychosocial Dementia Research Group within the authors' affiliated university, INTERDEM Academy [41] and peers and attendees of conferences related to dementia. The preliminary and final results were also disseminated to people with dementia during these conferences and their feedback was incorporated into the final report of this SCA.

## 3 Results

### 3.1 Overview of the Results

#### 3.1.1 Selection of Sources of Evidence

**FIGURE 1:**
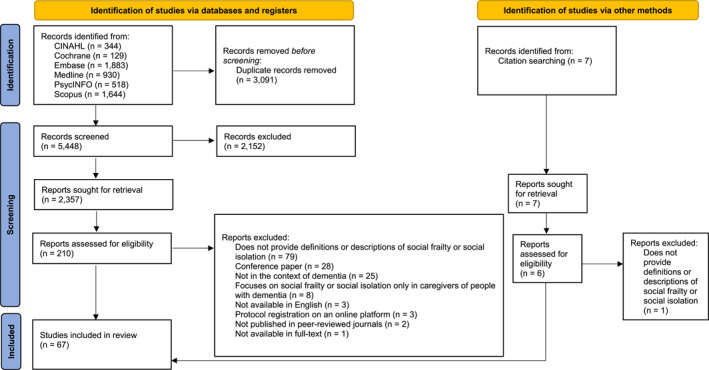
The Reporting Items for Simultaneous Concept Analysis flow diagram includes searches of databases and other sources. *Source:* M. J. Page, J. E. McKenzie, P. M. Bossuyt, I. Boutron, T. C. Hoffmann, C. D. Mulrow, et al. “The PRISMA 2020 Statement: An Updated Guideline for Reporting Systematic Reviews,” BMJ 372 (2021): n71, 10.1136/bmj.n71. For more information, visit: http://www.prisma‐statement.org/.

A total of 5445 publications were identified for title and abstract screening. The full texts of 217 publications were examined, from which 67 publications were selected for data. The process is detailed in a flow diagram (Figure 1) [42].

#### 3.1.2 Characteristics of the Included Publications

##### 3.1.2.1 Type of Evidence and Setting

The majority of studies were cross‐sectional (*n* = 17) [12, 20, 43, 44, 45, 46, 47, 48, 49, 50, 51, 52, 53, 54, 55, 56, 57], secondary analyses of datasets (*n* = 14) [13, 17, 26, 58, 59, 60, 61, 62, 63, 64, 65, 66, 67, 68], semi‐structured and structured interviews (*n* = 6) [69, 70, 71, 72, 73, 74] or longitudinal studies (*n* = 5) [75, 76, 77, 78, 79]. The remaining studies included various other types of research studies and reviews, such as quasi‐experimental studies or systematic reviews (*n* = 25) [16, 80, 81, 82, 83, 84, 85, 86, 87, 88, 89, 90, 91, 92, 93, 94, 95, 96, 97, 98, 99, 100, 101, 102, 103].

Most studies were conducted in community settings (*n* = 32), followed by nursing homes or residential settings (*n* = 14), and hospital or clinic settings (*n* = 5). A few studies reported multiple settings (*n* = 2), and the remaining did not report specific settings (*n* = 14).

##### 3.1.2.2 Characteristics of the Population of Participants

Many publications involved people diagnosed with dementia (*n* = 18), people living with or without dementia combined (*n* = 6) and people at risk for dementia (*n* = 3). Other specific groups included those with early‐stage dementia (*n* = 1), mild or mild to moderate stage dementia (*n* = 3), mildly severe to very severe dementia (*n* = 3) and LGBT (lesbian, gay, bisexual and transgender) people with dementia (*n* = 1). The remaining 32 papers had people without dementia at the baseline of a longitudinal study (*n* = 17), with some not reporting population characteristics (*n* = 15).

##### 3.1.2.3 Reporting Disciplines of Authors

Publications were often from multidisciplinary teams (*n* = 20), with authors from the background of nursing science (*n* = 10), health science or public health (*n* = 4), social science (*n* = 4), psychiatry (*n* = 7), psychology (*n* = 2) and neurology (*n* = 4) and the information systems (*n* = 1).

##### 3.1.2.4 Period and Number of the Included Publications

The publications span the years 1990–2024. Publications reporting on social frailty were from 2017 to 2023 (*n* = 10), while those reporting on social isolation were from 1990 to 2024 (*n* = 57). In the recent five years, there were five publications on the concept of social frailty, while 25 on social isolation. The significant difference in the number of publications suggests that, compared to social isolation, social frailty is a relatively new concept and not widely explored in dementia care and research. A table summarising the characteristics of the included 67 publications is provided in Supporting Information S2: Appendix 2.

### 3.2 Clarification of Individual Concepts and Validity Matrices of Social Frailty and Social Isolation

#### 3.2.1 Overview of Conceptual Definitions, Operational Definitions and Narrative Descriptions

Ten publications were analysed for definitions and descriptions of social frailty, and 57 publications on social isolation. Overall, four publications discussed both social frailty and social isolation.

Both concepts of social frailty and social isolation shared a common theme of reduced social interactions or engagement. Based on the conceptual definitions, social frailty can broadly include the elements of resource insufficiency and unfulfillment of social needs, and operational indicators of social frailty include financial difficulty. By contrast, social isolation is primarily described as reduced social networks, with operational measures that focus on the quality and quantity of social contact. From the narrative descriptions of people with dementia, social frailty is often expressed through loneliness and poor social contact. Similarly, social isolation meant having fewer friends, lacking closing relationships, being socially disconnected, living alone and being ignored (Table 1).

**TABLE 1:** Definitions and descriptions of social frailty and social isolation.

Concepts	Conceptual definitions	Operational definitions	Narrative descriptions
Social frailty	Can define as(*n* ^a^ = 5):	Can be operated as(*n* = 5):	Can be described as(*n* = 1):
	Less or lack of social activities or social engagement (*n* ^a^ = 4)	Living alone (*n* = 3)	Loneliness (*n* = 1)
	Loss or absence of social resources (*n* = 3)	Less talk (*n* = 4)	Poor social contact (*n* = 1)
	Less or lack of self‐management abilities (*n* = 3)	Inability to help others (*n* = 4)	
	Failure to fulfil basic social needs (*n* = 3)	Financial difficultly (*n* = 2)	
	Social functioning aspect of multidimensional frailty (*n* = 2)	Visiting friends less (*n* = 3)	
	Lack of social interactions or behaviour (*n* = 2)	Less frequently going out (*n* = 3)	
		Less contact with others: Friends and relatives (*n* = 2)	
		Lack of engaging in social activities (*n* = 2)	
		Loneliness (*n* = 2)	
		Less or absence of friends or relatives that feel close to asking for help (*n* = 2)	
Examples	Social frailty is a continuum of being at risk of losing or having lost, social and general resources, activities or abilities that are important for fulfilling basic social needs [22].	We operationalized social frailty using five questions from the baseline survey, including going out less frequently compared with the previous year, not visiting friends sometimes, not feeling helpful to friends or family, living alone and not talking with someone every day [54].	…Some aspects of psychosocial frailty, by poor social contacts … and loneliness [52].
Social isolation	Can define as (*n* = 16):	Can be operated as(*n* = 37):	Can be described as(*n* = 5):
	Reduced or lack of engaging in social activities (*n* = 7)	Less monthly social contact: Families, friends, neighbours, clubs (*n* = 20)	Being ignored by others (*n* = 1)
	Lack of social networks or social relationships (*n* = 8)	Less or absence of friends or (families) relatives that feel close to asking for help (*n* = 13)	Less friends (*n* = 1)
	Objective state (compared with the subjective state of loneliness) (*n* = 7)	Living alone (*n* = 11)	Living alone (*n* = 1)
		No married or partner (*n* = 6)	
	Social disconnectedness (*n* = 6)	Feeling apart from others (*n* = 12)	No close relationships (*n* = 1)
	Infrequent or lack of social contact with others (*n* = 7)	Limited participation in social activities (*n* = 12)	Social disconnectedness (*n* = 1)
	Limited or lack of social interaction (*n* = 4)	Less monthly visiting friends or families (visits) (*n* = 11)	
	Objective physical separation from others (*n* = 3)	Negative changes to social networks: Small networks (*n* = 9)	
		Feel lonely (*n* = 7)	
		Less monthly social interactions (*n* = 4)	
		Feel useless or less competent (*n* = 3)	
Examples	Social isolation is defined as an ‘*objective state of having few social relationships or infrequent social contact with others’* [13].	Social isolation was evaluated using seven criteria that assess contact with others and social participation: (1) lives alone, (2) has no close relatives, (3) never calls/writes anyone, (4) has no personal contact with neighbours/residents, (5) is alone for more than 9 h a day, (6) never goes out of the house/retirement home and (7) does not participate in neighbourhood, religious, political, social or retirement home activity [45].	‘*… Isolation … can be the dropping off of friends. They don’t get asked to things because, well, they’ve got dementia’* [70].

^a^ Number of publications reported.

#### 3.2.2 Clarification for Attributes of Social Frailty and Social Isolation

Sixteen attributes were identified for social frailty from 61 constructs across eight publications, and 17 for social isolation from 183 constructs across 42 publications (Table 2).

**TABLE 2:** Validity matrices for attributes, antecedents and consequences of social frailty and social isolation.

	Common factors	Social frailty	Social isolation
Attributes	Being alone	Living alone (*n* ^a^ = 3)	Living alone (*n* = 11)
		—	No married or partner (*n* = 6)
		—	Objective physical separation from others (*n* = 3)
		—	Feeling apart from others (*n* = 12)
		—	Being ignored by others (*n* = 1)
	Limited or less social activities, engagement or interactions	Less or lack of social activities or social engagement (*n* = 4)	Reduced or lack of engaging in social activities (*n* = 16)
		Lack of social interactions or behaviour (*n* = 2)	Limited or lack of social interaction (*n* = 4)
		—	Less monthly social interactions (*n* = 4)
		Less frequently going out (*n* = 3)	—
		—	Social disconnectedness (*n* = 7)
	Less or absence of social resources and social support	Less or absence of friends or relatives that feel close to asking for help (*n* = 2)	Less or absence of friends or (families) relatives that feel close to asking for help (*n* = 14)
		Loss or absence of social resources (*n* = 3)	—
		Failure to fulfil basic social needs (*n* = 3)	—
	Less social contact and visits	Less contact with others: Friends and relatives (*n* = 2)	Less monthly social contact: Families, friends, neighbours, clubs (*n* = 20)
		Poor social contacts (*n* = 1)	Infrequent or lack of social contact with others (*n* = 7)
		Visiting friends less (*n* = 3)	Less monthly visiting friends or families (visits) (*n* = 11)
	Loneliness	Loneliness (*n* = 3)	Feel lonely (*n* = 7)
	Limited self‐management abilities or competence	Less or lack of self‐management abilities (*n* = 3)	Feel useless or less competent (*n* = 3)
		Inability to help others (*n* = 4)	—
	Component/state	Social functioning dimension of multidimensional frailty (*n* = 2)	Objective state (compared with the subjective state of loneliness) (*n* = 7)
		Financial difficultly (*n* = 2)	—
		Less talk (*n* = 4)	—
		—	Lack of social networks or social relationships (*n* = 15)
Antecedents	Age‐related factors	Ageing (*n* = 1)	Age‐related hearing loss (*n* = 4)
	Being alone	Living alone (*n* = 1)	Exclusion from others (*n* = 2)
		No married or partner (*n* = 1)	
	Lack of social activities	Lack of social activities (*n* = 1)	Limited participation in social activities (*n* = 2)
		—	Lack of meaningful activities (*n* = 2)
		—	Less frequently going out (*n* = 2)
		Impact of socio‐economic welfare (*n* = 1)	—
		—	Cognitive decline (*n* = 5)
		—	Dementia diagnosis and symptoms (*n* = 12)
		—	Less contact and lack of communication (*n* = 9)
		—	No inclusive residential care services (*n* = 7)
		—	Negative public attitudes, stigma and discrimination (*n* = 4)
		—	Smaller social networks (*n* = 4)
		—	Decreased or absence of engaging in social interactions (*n* = 4)
Consequences	Cognitive decline	Yes (*n* = 5)	Yes (*n* = 6)
	Increased risk for dementia	Yes (*n* = 3)	Yes (*n* = 9)
	Negative psychological effects	Depressive symptoms and depression (*n* = 1)	Depression (*n* = 4)
		—	Adverse mental health (*n* = 5)
		—	Increase stress and anxiety (*n* = 2)
	Negative effects on quality of life	Harmful effects on quality of life (*n* = 2)	Decreased satisfaction with quality of life (*n* = 2)
	Physical and functional effects	Generally and physically functional decline (*n* = 2)	Loss of function (*n* = 2)
		—	Adverse physical health (*n* = 4)
	Negative health‐related outcomes	Increased risk for negative health outcomes (e.g., disability, mortality.) (*n* = 2)	Increased risk for negative health outcomes (e.g., mortality.) (*n* = 8)
		Physical frailty (*n* = 5)	—
		Psychological frailty (*n* = 1)	—
		—	Decreased access to social resources (*n* = 2)
		—	Lower social well‐being (*n* = 5)
		—	Feel lonely (*n* = 3)

^a^ Number of publications reported.

Both concepts of social frailty and social isolation share common factors such as living alone and experiencing loneliness. Significant overlaps of attributes between social frailty and social isolation exist in limited or less social activities, engagement or interactions, less social support from friends or relatives, infrequent social contact and visits and limited self‐management abilities or competence.

Attributes specific to social frailty include financial difficulty and less talk, which serve as operational definitions of the concept. Less frequently going out, a lack of social resources and failure to fulfil basic needs, are also distinctive attributes of social frailty.

Distinctive attributes of social isolation include a lack of social networks or social relationships. The attributes categorised under ‘being alone’ include not being married or having a partner, objective physical separation from others, feeling apart from others and being ignored by others. When compared with social frailty, the attributes of social isolation provide more details about the nature of social isolation rather than some indicators such as being alone or lack of social activities.

#### 3.2.3 Clarification for Antecedents of Social Frailty and Social Isolation

Five antecedents were identified from six constructs across one publication for the concept of social frailty, and 12 antecedents were identified across 31 publications for social isolation (Table 2).

The concept of social frailty and social isolation shared several antecedents including age‐related factors, being alone, and lack of social activities. Ageing was an antecedent for social frailty, while the publications on social isolation specifically reported age‐related hearing loss as an antecedent. Being alone was a shared construct for both social frailty and social isolation. Contributing constructs for this were living alone, not being married or having a partner for social frailty, and exclusion from others for social isolation. Lack of social activities was described as an antecedent of both social frailty and social isolation, while social isolation also highlighted meaningful activities besides the quantity of social activities.

Impact of socio‐economic welfare was the only reported construct as an antecedent of the onset of social frailty. Dementia diagnosis and symptoms, less contact and lack of communication, no inclusive residential care services, and cognitive decline, followed by negative public attitudes, smaller social networks and decreased social interactions were distinct antecedents of social isolation.

#### 3.2.4 Clarification of Consequences of Social Frailty and Social Isolation

Eight consequences were identified for social frailty from 31 constructs across eight publications and 11 for social isolation from 56 constructs across 19 publications (Table 2).

Consequences such as cognitive decline, increased risk for dementia, physical and functional effects and negative health‐related outcomes overlapped in both social frailty and social isolation. Additionally, depressive symptoms were reported as consequences of both concepts, while literature on social isolation also reported adverse mental health and increased stress and anxiety highlighting its negative psychological effects. Negative effects on quality of life were also identified as consequences of both concepts in terms of (with links to) harmful effects on quality of life or decreased satisfaction with quality of life.

Other aspects of frailty, such as physical frailty and psychological frailty, were reported as distinct constructs regarding consequences of social frailty. Feeling lonely and having reduced social resources and well‐being were distinct to the concept of social isolation as the consequences of it.

**FIGURE 2:**
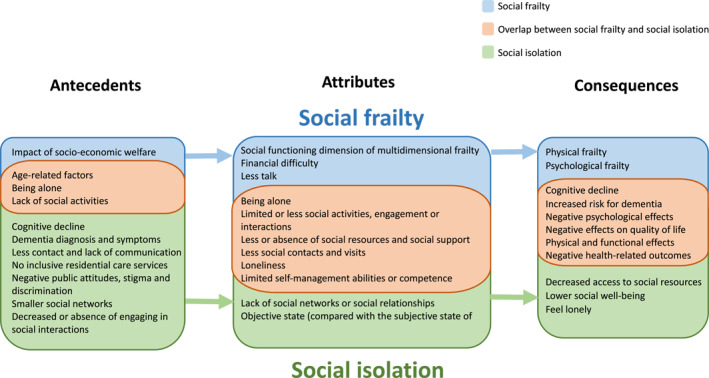
Process model of social frailty and social isolation. *: A process model serves as a tool for analysis and a predecessor to theories, not to be a theoretical or causal model.

Antecedents, attributes, and consequences of each concept of social frailty and social isolation in relation to one another are provided in the process model (Figure 2).

## 4 Discussion

This simultaneous concept analysis (SCA) examined conceptual definitions, operational definitions and narrative descriptions of concepts of social frailty and social isolation. It also explored similarities and differences between antecedents, attributes and consequences of two concepts, and analysed their relationship in the context of dementia. The detailed exploration of social frailty and social isolation enhanced the understanding of the conceptual framework for social health of people with dementia [8], where social health is both an outcome and vital part of managing dementia, emphasising the need for addressing the social health risks of people with dementia.

Although the number of publications on the two concepts of social frailty and social isolation included in this SCA is quite varied, similarities and differences exist in the definitions, descriptions, attributes, antecedents and consequences of each concept. In this SCA, only 10 publications were included for the analysis of social frailty while 57 publications were included for analysing the concept of social isolation. Social frailty, which is suggested as a relatively recent concept within dementia research, has drawn little academic interest and has only started to appear in the literature since 2017 [12]. In contrast, social isolation has been extensively explored and well‐studied both for definitions [24] and measurement tools [43, 87]. Other studies on social isolation such as influencing factors [84], associations with dementia incidence [63] and intervention programmes and their impact on individuals living with dementia [72] also support the extensive body of research on this concept. This disparity indicates that social frailty is a less mature concept than social isolation, which requires further investigation in the context of dementia specifically regarding its application and social health outcomes in dementia populations.

The publications included in this SCA highlighted the interlinked nature of the two concepts of social frailty and social isolation, demonstrating different facets of each concept. Both concepts overlap in several aspects of their attributes, primarily in ‘limited or less social activities, engagement or interactions’ both on the conceptual and operational definitions of the two concepts [16, 17, 54, 76]. Social frailty uniquely includes operationalised items such as ‘less frequently going out’ [54], while ‘engagement in social activities’ is a definition and measurement item of social isolation [86]. Furthermore, social isolation has also been referred as a degree of social disconnection [84] and inadequate quality and quantity of social connectedness [57]. Social interactions refer to the direct acts of communication or engagement with others [104], while social connectedness is a broader term that includes living arrangements, size of social networks, and engagement in social activities [105]. This suggested the concept of social frailty focuses only on the quantity of engaging in social activities or interactions, whereas social isolation encompasses a broader range of measurements, focussing on both the quantity and quality of social connectedness not only interactions.

Publications included in this SCA suggest that social frailty involves a one‐way reduction or absence in the attribute ‘visiting friends less’ [95] indicating that the number of visits to friends is reduced due to the reasons related to the person with dementia. Studies suggested that such people with social frailty have more impaired ability in terms of verbal fluency [106] and have issues with hearing impairment [107], which might affect their desire, interest, or capacity to visit friends. This reflects another unique attribute ‘less talk’ [54], which was also an item in most measurement tools for social frailty. In contrast, social isolation is suggested to refer to more of a reduction or absence in two‐way activities, for example, both visiting friends and being visited by friends [63]. This highlights a much broader impact extending beyond the socially isolated individuals' actions. It might also reflect the distinctive attribute ‘lack of social networks and social relationships’ [15]. Individuals experiencing social isolation reported that being ignored by others contribute to reduced interactions with their social networks, leading to fewer visits by others [73]. Additionally, having ‘smaller social networks’ [69] is identified as a distinctive antecedent of social isolation, suggesting that attributes and antecedents may overlap within the concept of social isolation.

Although both concepts involve less or an absence of social support from friends, families or relatives, the attributes of social frailty distinctly reflect an absence of broader social resources and financial difficulty [16, 17, 95]. Social resources refer to resources or services that individuals with dementia and their family caregivers can apply for, such as day care, home help services, responsive care and home care, which can provide additional support to them [108, 109]. Socially frail people with dementia might retire early from their employment due to dementia symptoms [110], which could to economic challenges and lack of access to healthcare or other essential services they need. The lack of social resources might often intersect with economic challenges, highlighting how financial difficulty directly affects an individual's ability to engage with and access required social resources. Limitations with the welfare system might also lead socially frail individuals to financial difficulties [111]. This reflects the distinctive antecedent ‘impact of socio‐economic welfare’ of social frailty [95]. Financial difficulty as one of the distinctive attributes of social frailty can be an important consideration under the socio‐economic factors [95] when developing or adapting measurement tools for social frailty and informing the development of prevention programmes for social frailty in the dementia population.

**FIGURE 3:**
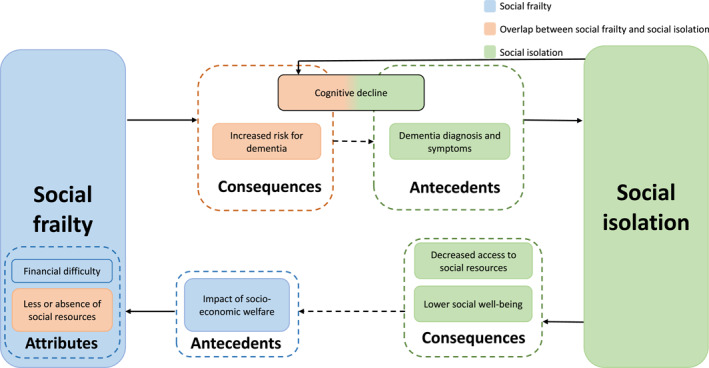
Examples of antecedents, attributes, and consequences that link and differentiate the concepts of social frailty and social isolation. *The figure represents the interrelated and mutual influences between both concepts together with their consequences and antecedents.

‘Decreased access to social resources’ [84] and having ‘lower social well‐being’ [90] are identified as distinctive consequences of social isolation. People with dementia who are experiencing social isolation are more likely to experience poorer health outcomes than those who are not [112], possibly due to increasingly lacking access to essential social resources and having reduced social well‐being. As social isolation advances, the reduced access to social resources and lower social well‐being potentially contribute to social frailty. This indicates that attributes of social frailty and consequences of social isolation overlap, showing while both concepts are related, each has its distinct pathways and impacts. The relationship between this attribute of social frailty and consequence of social isolation might suggest that social frailty and social isolation are interrelated and mutually influence each other. Figure 2 displays distinct and overlapping antecedents, attributes, and consequences of both concepts. Figure 3 presents a selection of examples to illustrate how these antecedents, attributes, and consequences link and differentiate the concepts of social frailty and social isolation. We emphasise that the connections we show between the two concepts and their antecedents, attributes, and consequences should not necessarily be interpreted as causal pathways. Our results highlight the importance of early management of dementia and social frailty by enhancing social resources and decreasing the risks and effects of social isolation. By addressing such issues, interventions can be more effectively designed to target social health outcomes for people with dementia.

Based on the available literature, ‘cognitive decline’ and ‘dementia symptoms and diagnosis’ act as antecedents only for the concept of social isolation. This suggests that the onset of cognitive decline and dementia might lead to individuals' social isolation. Cognitive decline may reduce an individual's ability to engage in social interactions due to memory loss [67], and lead to challenges in communication [68, 74]. Similarly, the diagnosis of dementia might contribute to social isolation due to changes to social networks [72], potentially linking with stigma or discrimination from public [69, 73]. On the other hand, ‘cognitive decline’ and ‘increased risk for dementia’ are recognised as consequences of both social frailty and social isolation [54, 61, 65, 99]. This indicates a relationship between both concepts, due to overlapping antecedents and consequences related to dementia risk, symptoms and diagnosis (Figure 3). The concepts of social frailty and social isolation have been used interchangeably in previous research [9, 19, 20, 27, 28, 29, 30, 31, 32], and this SCA identified some overlaps between these concepts; however, although they are interrelated, they are not identical. This suggests that managing social frailty might prevent individuals' cognitive decline and reduce the risk of dementia. It could also be possible to both prevent social isolation by managing cognitive decline and to prevent further progression of cognitive decline and decrease the risk for dementia by managing social isolation. This can be addressed by development of prevention and management programmes targeting both social frailty and social isolation in the dementia population and those at risk. Additionally, it may help the recognition of the unique challenges faced by those with early onset dementia, who may experience social frailty or social isolation at a younger, potentially more active stage in their lives. Managing social frailty and social isolation in early‐onset dementia might also potentially slow the progression of their disease. Understanding the unique challenges experienced by this population can aid in developing more targeted care strategies and services for people with dementia. This approach could help maintain the quality of life and improve health outcomes of people with dementia, potentially reducing inequalities in dementia population.

Examples of other concept analyses of social frailty [113] and social isolation [114] in older adults have been noted. A recent concept analysis of social frailty in the context of older adults [113] reported that low cognitive function, including dementia, is one of the psychological consequences of social frailty in older adults. This aligns with our analysis of ‘cognitive decline’ as a consequence of social frailty in the context of dementia. It suggests that social frailty should be managed in older adults to delay cognitive decline in order to prevent dementia onset. Another concept analysis, which was about social isolation in older adults [114] also described dementia and decline in cognition as possible causes of social isolation in the older population, which also supports our results identifying ‘cognitive decline’ and ‘increased dementia risk’ as consequences of social isolation in the context of dementia. Our SCA further added to the existing knowledge by simultaneously analysing both concepts of social frailty and social isolation and specifically exploring these concepts in the context of dementia.

Potential limitations to this SCA include interpretive bias during the categorisation of conceptual and operational attributes of each concept, however, this simultaneous concept analysis process followed guidelines outlined by Méndez‐Sánchez [115] and was reviewed and discussed by the consensus group members through regular meetings. The common factors and distinctive attributes, antecedents, or consequences of both concepts in this SCA are also limited by the quantity of publications, particularly concerning social frailty. It is important to note that the disparate number of publications on social frailty and social isolation could have influenced the identification of distinctive attributes, antecedents or consequences. It is also recognised as a limitation that non‐English publications were excluded, thereby potentially omitting some relevant evidence. Another limitation is that people with dementia and clinical practitioners were not directly involved in the development of this SCA. However, they were part of the process by providing feedback on the preliminary and final results.

Despite the limitations listed above, this SCA has several strengths. Following a comprehensive review and synthesis of the literature, this SCA is the first to identify the distinct and overlapping attributes, antecedents, and consequences of both social frailty and social isolation in the context of dementia, thereby enhancing the clarity of definitions and elucidating their interrelationships. Additionally, this SCA is the first to explore both the differences and similarities between these two important concepts. This SCA also included publications that were longitudinal studies including baseline populations without dementia and their results contributed to the development of antecedents, attributes, and consequences of both concepts. We focussed on both of these population groups to identify the interrelationships in the literature and present the broader picture of social frailty and social isolation in populations at pre and post diagnosis stages of dementia. The results of this SCA will inform the development of more targeted and effective strategies to address social frailty and social isolation in people with dementia, contributing significantly to both academic and practical advancement in the field of dementia care. Future studies should evaluate the results of this simultaneous concept analysis using relevant primary data collection methods [33]. This SCA also highlighted the need for more publications particularly concerning social frailty in dementia.

## 5 Conclusion

This publication supports the use of SCA and the development of more comprehensive social frailty and social isolation measurement tools for use with people with dementia. Furthermore, the findings from this study can be used to better identify people with dementia at risk of social frailty or social isolation as well as guide future researchers and policymakers to develop social frailty or social isolation prevention and management programmes, thereby improving the social health of people with dementia.

## Author Contributions

**Ziyue Wang:** conceptualization (lead), funding acquisition (lead), methodology (lead), project administration (lead), writing – original draft preparation (lead), writing – review and editing (equal). **Dympna Casey:** conceptualization (equal), methodology (supporting), supervision (supporting), writing – review and editing (supporting). **Duygu Sezgin:** conceptualization (equal), funding acquisition (supporting), methodology (supporting), supervision (lead), writing – original draft preparation (supporting), writing – review and editing (equal).

## Ethics Statement

This study represents secondary research and did not require ethical approval.

## Conflicts of Interest

The authors declare no conflicts of interests.

## Supporting information

Supporting Information S1

Supporting Information S2

## Data Availability

The data that support the findings of this study are openly available in Open Science Framework at https://osf.io/n586a, reference number https://doi.org/10.17605/OSF.IO/N586A.
