# Supplementary material for: Social Frailty and Social Isolation in the Context of Dementia: A Simultaneous Concept Analysis
Source: Int J Geriatr Psychiatry. 2025 Apr 19;40(4):e70074. doi: 10.1002/gps.70074 (PMC12009061; doi:10.1002/gps.70074)
Supplement: Supplementary file 1 — Supporting Information S1 [file GPS-40-e70074-s002.docx]

**Appendix 1. The search strategy and database search results**

| Databases | Searching strategies | Results |
| --- | --- | --- |
| PubMed | ( ("social frailty"[Text Word] OR "social frail"[Text Word]) AND ("Alzheimer disease"[Text Word] OR "Alzheimer*"[Text Word] OR "Dementia"[Text Word] OR "dement*"[Text Word] OR "Dementia"[MeSH Terms] OR "Alzheimer disease"[MeSH Terms])) OR ( ("social isolation"[MeSH Terms] OR "social isolation"[Text Word]) | 1150 |
| Ovid Medline | (("social frailty".mp.) AND ((Dementia or Alzheimer Disease or (Dementia or Alzheimer Disease)).mp.))OR ((Social Isolation or Social Isolation).mp.) | 930 |
| CINAHL | ''social frailty'' AND (MH Alzheimer's disease OR Alzheimer* OR MH dementia OR dementia) OR (MH social isolation OR MH Social Isolation (Saba CCC)/ET/EV/NU/TH/ED/UT OR MH Social Isolation (NANDA)/ET/EV/UT) OR social isolation) | 344 |
| PsycINFO | ''social frailty'' OR (SU social isolation OR social isolation) AND (SU dementia OR dementia OR Alzheimer* OR SU Alzheimer’s disease) | 518 |
| Scopus | ( TITLE-ABS-KEY ( "social isolation" ) ) OR (TITLE-ABS-KEY ( "social frailty" ) ) AND ( ( TITLE-ABS-KEY ( dement* ) OR TITLE-ABS-KEY ( dementia ) OR TITLE-ABS-KEY ( Alzheimer* ) OR TITLE-ABS-KEY ( "Alzheimer’s disease" ) ) ) | 1644 |
| Embase | ('social frailty'/exp OR 'social frailty' OR 'social frail*') OR ('social isolation'/exp OR 'social isolation':ti,ab,kw) AND ('dementia'/exp OR 'Alzheimer disease'/exp OR Alzheimer* OR dement*) | 1883 |
| Cochrane Library | (''social frailty''):kw OR (''social isolation''):kw AND (MeSH descriptor: [Dementia] this term only OR MeSH descriptor: [Alzheimer Disease] this term only OR (dementia):kw OR (''Alzheimer*''):kw) | 129 |
